# Investigations of structural and dynamical mechanisms of ice formation regulated by graphene oxide nanosheets

**DOI:** 10.1063/4.0000111

**Published:** 2021-09-14

**Authors:** Shengkai Zhang, Jingjing Han, Xiang Luo, Zhixin Wang, Xudong Gu, Na Li, Nicolas R. de Souza, Victoria Garcia Sakai, Xiang-Qiang Chu

**Affiliations:** 1 Institute of High Energy Physics, Chinese Academy of Sciences, Beijing, China; 2 Graduate School of China Academy of Engineering Physics, Beijing, China; 3 University of Chinese Academy of Science, Beijing, China; 4 National Facility for Protein Science in Shanghai, Zhangjiang Laboratory, Shanghai Advanced Research Institute, CAS, No.333, Haike Road, Shanghai 201210, China; 5 Australian Nuclear Science and Technology Organization, Lucas Heights, NSW 2234, Australia; 6 ISIS Facility, Rutherford Appleton Laboratory, Chilton, Didcot OX11 0QX, United Kingdom

## Abstract

Recent research indicates that graphene oxide (GO) nanosheets can be used to regulate ice formation by controlling critical ice nucleus growth in water at supercooling temperatures. In addition, the study of ice formation mechanisms regulated by GO nanosheets, a good model system for antifreeze proteins (AFPs), will shed light on how AFPs regulate ice formation in nature. In this work, time-resolved small-angle x-ray scattering (TR-SAXS) and quasi-elastic neutron scattering (QENS) experiments were carried out to investigate the structural and dynamical mechanisms of ice formation regulated by GO nanosheets. Strikingly, a transient intermediate state was observed in TR-SAXS experiments that only exists in the aqueous dispersions with a larger GO size (11 nm). This serves as evidence that the size of GO is critical for regulating ice formation. Elastic neutron scattering results indicate that ice is formed in all samples and thermal hysteresis occurs in GO aqueous dispersions in both H_2_O and D_2_O. The structural and dynamics information about water molecules in GO, extracted from QENS, reveals different dynamical behaviors of water molecules in GO aqueous dispersions when approaching the ice formation temperature.

## INTRODUCTION

Nature has unique ways of regulating ice formation; for example, antifreeze proteins (AFPs) protect organisms from freeze damage by regulating the formation of ice^1^ via controlling the arrangement of hydroxyl groups. Ice nucleation is the controlling step in water freezing,^2–6^ and it has long been assumed to require the formation of a critical ice nucleus.^4–11^ Direct experimental evidence for the existence of such a critical ice nucleus has been observed recently in water droplets containing graphene oxide (GO) nanosheets.^12^ The GO nanosheets in water droplets have a notable impact on ice nucleation only above a certain size that varies with the degree of supercooling of the droplets. Both experimental results^13^ and theoretical calculations^14^ have shown the coexistence of large oxidized and unoxidized graphene regions on the surface of GOs. Hydroxy(-OH) and epoxy(-O-) groups are located at oxidized regions of the basal plane of GOs, whereas the carboxyl groups mainly localize at the periphery of GOs.^15^ The plane of GO consists of repeat honeycomb hexagonal carbon rings, forming a scaffold structure to arrange the hydroxy groups on GOs and facilitate the ice crystal lattice matching. It is reminiscent of the hydroxy group organization on the ice-binding surface of AFPs. Previous study indicates that GOs induce a thermal hysteresis (TH, the difference between the equilibrium melting and freezing temperatures of an ice crystal), which is a typical feature shared with all AFPs.^16^ This implies that GOs may mimic AFPs in controlling ice formation. Therefore, systematic study of the GO nanosheets regulated ice formation mechanisms has the potential to give us comprehensive understanding of how AFPs regulate ice formation in nature.

In this study, we use time-resolved small-angle x-ray scattering (TR-SAXS) and quasi-elastic neutron scattering (QENS) to investigate the structural and dynamical mechanisms of ice formation regulated by GO nanosheets, respectively. GOs with two different sizes critical for ice formation were used in the experiments. A transient intermediate state was observed in time series scattering profiles, however only in aqueous dispersions with the larger GO size (11 nm). TR-SAXS results provide additional experimental evidence that the size of GO is critical for regulating ice formation. QENS has been used to selectively characterize the motion of water in the graphene oxide aqueous dispersion, owing to the much larger incoherent scattering cross section of hydrogen atoms compared to that of all other atoms in the system. Aqueous dispersions of both H_2_O and D_2_O were studied, and a thermal hysteresis (TH) was observed from the elastic neutron scattering (ENS) intensities. Detailed QENS measurements were taken to study the dynamics of water molecules in the GO aqueous dispersions at temperatures above and approaching ice formation, and results analyzed in both the energy and time domains.

## MATERIALS AND METHODS

### Sample preparation

GOs with average lateral sizes of 8 and 11 nm were prepared according to the method reported in Ref. 12 by fractionating commercial GO aqueous dispersions by consecutively filtering through ultrafiltration membranes (Ultracel) with different molecular weight cutoffs. Molar concentrations of GO aqueous dispersions were estimated from their mass concentrations and the molar mass of GOs. The details are described in Ref. 34.

### Time-resolved small-angle x-ray scattering

The time-resolved SAXS measurements were performed at BL19U2 beamline of the National Center for Protein Science Shanghai (NCPSS) at Shanghai Synchrotron Radiation Facility (SSRF). The wavelength range is 0.82–1.77 Å. The x-ray wavelength used in present study was set as 1.03 Å. Scattering intensities were collected with a Pilatus 1M detector (DECTRIS Led., Baden, Switzerland). The sample-to-detector distance was set as 2.6 m to cover a *Q* range of 0.008–0.4 Å^−1^ [*Q *=* *4*π*sin(*θ*)/λ, where 2*θ* is the scattering angle]. GO nanosheets with two sizes, 8 and 11 nm, in double-distilled water were used with the same mass fraction 0.05 wt. % (13 *μ*mol/l). Samples were kept in a quartz capillary with a diameter of 1.5 mm and a wall thickness of 10 *μ*m. The heating stage HFSX350 (Linkam Ltd., Tadworth Surrey, UK) was installed at the sample position for cooling experiments. The x-rays were focused to the detector plane with a focal size of 0.40 × 0.06 mm^2^ (H × V) at the sample position with a photon flux of 5 × 10^12^ photons/s. The heating stage enables rapid cooling followed by immediate exposure to x-ray measurement. The temperature of the heating stage drops from −12 °C to −30 °C at the cooling rate of 1 °C/min. The exposure time of the detector per frame was 0.1 s with an interval of 10 s. According to the setting of cooling and exposure interval time, there are in total 108 frames of SAXS data for 8 and 11 nm GO samples, respectively. The data reduction procedure includes correction according to the BL19U2 configuration file.^17–19^ In general, the 2D scattering images were converted to 1D SAXS curves through azimuthally averaging after solid angle correction and then normalizing with the intensity of the transmitted x-ray beam, using the software package BioXTAS RAW.^20^ Background scattering was subtracted using PRIMUS in ATSAS software package.^21^

### Quasi-elastic neutron scattering

QENS measurements were performed on the time-of-flight backscattering neutron spectrometer, IRIS, at the ISIS Neutron and Muon Source,^22–24^ UK, and on the cold neutron backscattering spectrometer, EMU, at the Open Pool Australian Lightwater (OPAL) research reactor at ANSTO,^25^ Australia. For the IRIS measurements, the energy resolution was 17.5 *μ*eV (full width at half maximum, for the Q-averaged resolution value) and the accessible dynamic range was ±500 *μ*eV. The crystal analyzer was pyrolytic graphite 002 corresponding to an (elastic) Q range of ∼0.42–1.85 Å^−1^. The samples were kept in cylindrical aluminum cans with 0.25-mm gap, and their effective volume was ∼1 ml. The raw data were normalized to incoming flux and vanadium, corrected for the contribution of empty cell and detector efficiency, and then transferred to energy and momentum transfer scales to obtain the scattering function S(Q,ω) using the program package MANTID.^26^ The measurements on Emu set up with silicon 111 crystal analyzers were conducted using a Q range of 0.35 Å^−1^ < Q < 1.95 Å^−1^, providing 1 *μ*eV full width at half maximum (FWHM) energy resolution for an accessible ±31 *μ*eV energy transfer range.

Elastic scans, where the elastic intensity is recorded as a function of temperature, were performed on four samples: 8 nm GOs and 11 nm GOs in H_2_O, and pure H_2_O and 8 nm GOs in D_2_O. The samples were first cooled from ambient temperature (∼300 K) down to ∼220 K at a ramp rate of 0.5 K/min and then warmed from 220 K up to ambient temperature at a rate of approximately 1 K/min.

For the QENS measurements, the 8 nm GO in H_2_O dispersion was measured at three temperatures, 300, 265, and 260 K, plus a low temperature measurement, at 9 K, was taken to use as the resolution function. The 8 nm GO in D_2_O was measured at 300 K, and the resolution function was measured at 6 K. In our measured Q range, no obvious Bragg peaks show up in the low temperature measurements, so they are readily used as resolution functions. All GO samples in the ENS and QENS measurements have a mass fraction of 3.5 wt. % in dispersion, enabling us to directly compare the experimental results of different samples, to demonstrate the size effect of GOs to H_2_O and the effect of GOs to the dynamics of H_2_O and D_2_O.

## RESULTS AND DISCUSSION

### Transient intermediate state observed by time-resolved SAXS

**FIG. 1.**
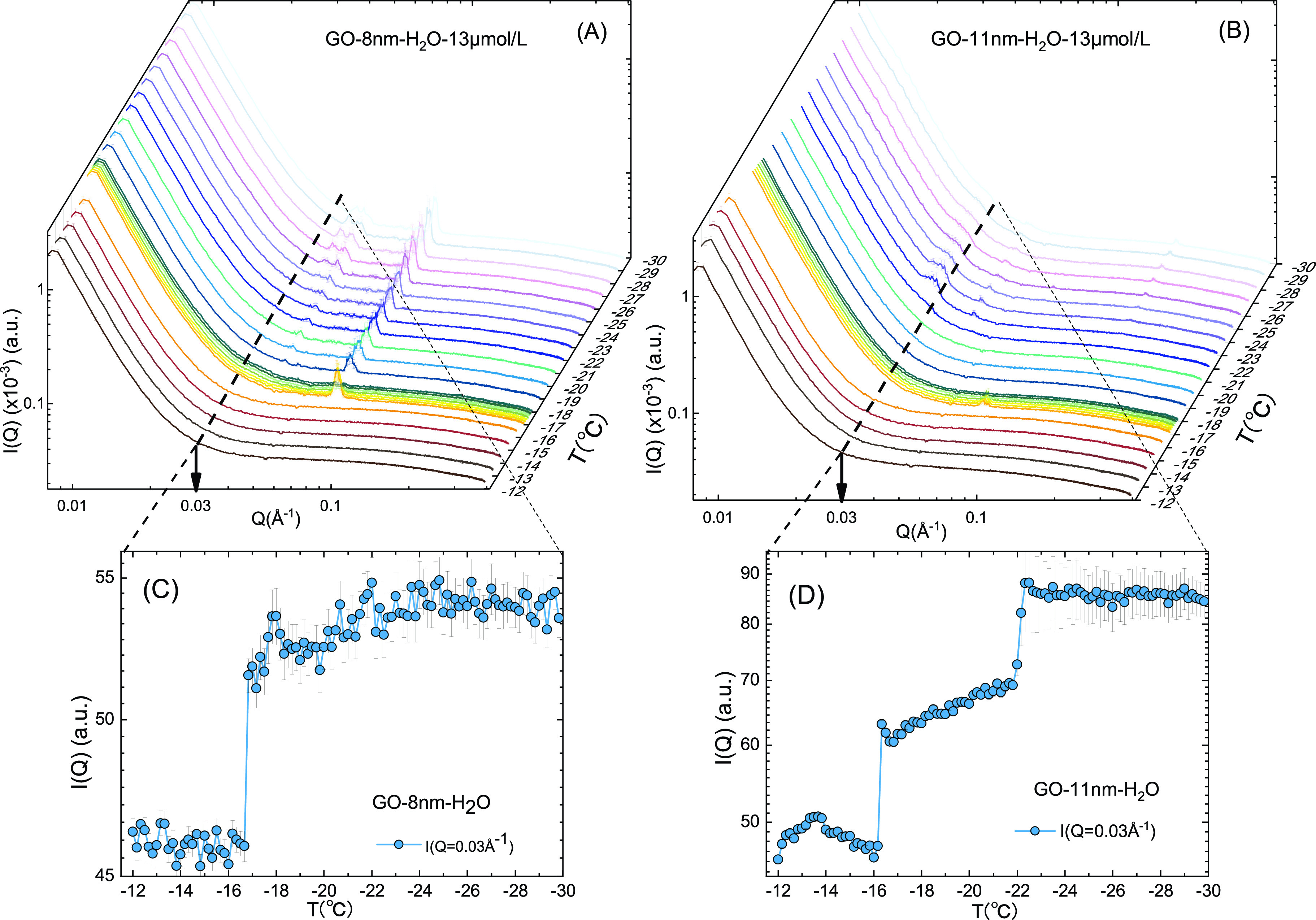
Time-resolved SAXS scattering intensity of (a) 8 nm and (b) 11 nm GO dispersions at concentration of about 13 *μ*mol/l in the temperature range of −12 °C to −30 °C at the cooling rate of 1 °C/min, measured every 10 s. The intensity data at Q = 0.03 Å^−1^ taken from curves in (a) and (b), plotted as functions of temperatures, for (c) 8 nm and (d) 11 nm GO samples, respectively. The apparent plateau in panel d implies the appearance of a transient intermediate state.

To capture the transient critical nucleus in ice formation, time-resolved small-angle x-ray scattering (TR-SAXS) experiments were designed to take measurements every 10 s during the cooling of the samples from −12 °C to −30 °C at the cooling rate of 1 °C/min. Here, we measured the aqueous dispersions with two different sizes of GOs (8 and 11 nm) at a concentration of ∼13 *μ*mol/l. The SAXS scattering intensities are plotted at the complete set of temperatures as functions of scattering wave vector Q, as shown in Figs. 1(a) and 1(b) for 8 and 11 nm GO aqueous dispersions, respectively. When the temperature decreases from −12 °C to −16 °C, the SAXS data of both samples remain unchanged. Below −17 °C, diffraction peaks start to appear, indicating ice formation in this supercooled temperature range. In contrast, in the 11 nm GO sample, no diffraction peaks were observed from −12 °C to −21 °C, and diffraction peaks were obvious below −22 °C [see Fig. 1(b)].

In Figs. 1(c) and 1(d), to better compare the difference between the two samples, we plot the SAXS intensity at a specific Q value (Q = 0.03 Å^−1^) as functions of temperature for the 8 and 11 nm samples, respectively. Here, we chose Q = 0.03 Å^−1^ to avoid Bragg peaks after ice formation at low temperatures, and the corresponding measuring length of this Q value is comparable to the size of GOs, which means the SAXS signals around this Q range reflect the structural information of the interface between water and GO nanosheets or ice nucleus. The comparison between the results from two samples indicates that the aqueous dispersion of 8 nm GOs was frozen in the temperature range from −17 °C to −22 °C, but the 11 nm GOs did not freeze. Strikingly, an apparent intermediate state can be observed in the case of 11 nm GOs [the plateau in Fig. 1(d)], but not in the 8 nm GOs [the abrupt transition at −17 °C in Fig. 1(c)]. Previous work reports that GO nanosheets dispersed in water droplets have a notable impact on ice nucleation only above a certain size (about 10 nm), which varies with the degree of supercooling of the droplets.^12^ When the GOs' sizes are smaller than that of the critical ice nucleus, pinning at the periphery of the GOs deforms the ice nucleus as it grows. Here, our observation is the formation of an intermediate state in the case of 11 nm GOs but not in 8 nm GOs, and the ice formation temperatures are higher than that reported in previous research.^12^ The possible reason is that, while we used capillary tubes in the SAXS experiments (Al cells in QENS experiments), previous measurements^12^ used GO nanosheets anchored on silicon wafer surfaces, excluding the possible influence on ice nucleation of GO diffusion and GO-GO interactions when dispersed in water.

### QENS experiments reveal dynamical process behind ice nucleation

**FIG. 2.**
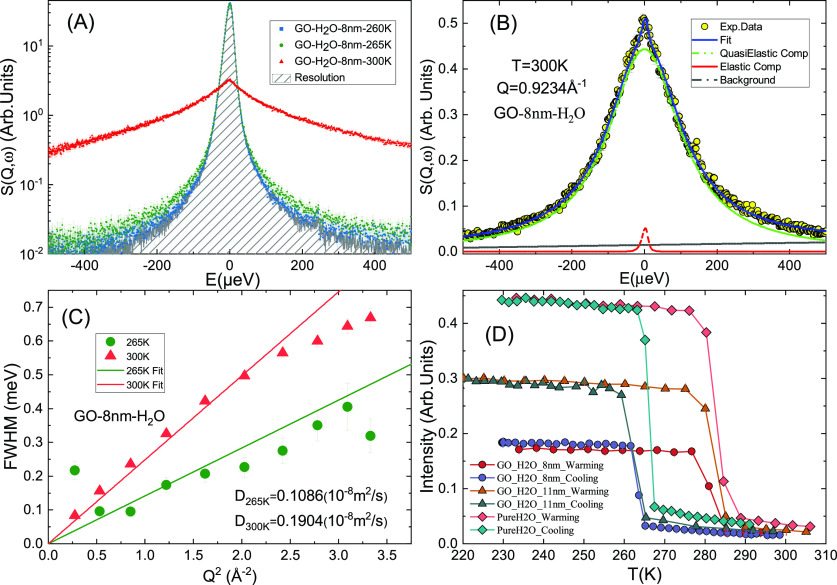
QENS data and analysis of 8 nm GOs in H_2_O dispersions at three different temperatures. (a) The normalized QENS spectra for 8 nm GOs in H_2_O at 260, 265, and 300 K covering the full Q range; (b) an example of model-independent analysis of QENS data at Q = 0.9234 Å^−1^; (c) the full width at half maximum (FWHM) 2Γ(Q) plotted as functions of Q^2^, fitted linearly to obtain the diffusion coefficient; and (d) the elastic scan intensity vs temperature plot, cases of warming and cooling for all three samples.

**FIG. 3.**
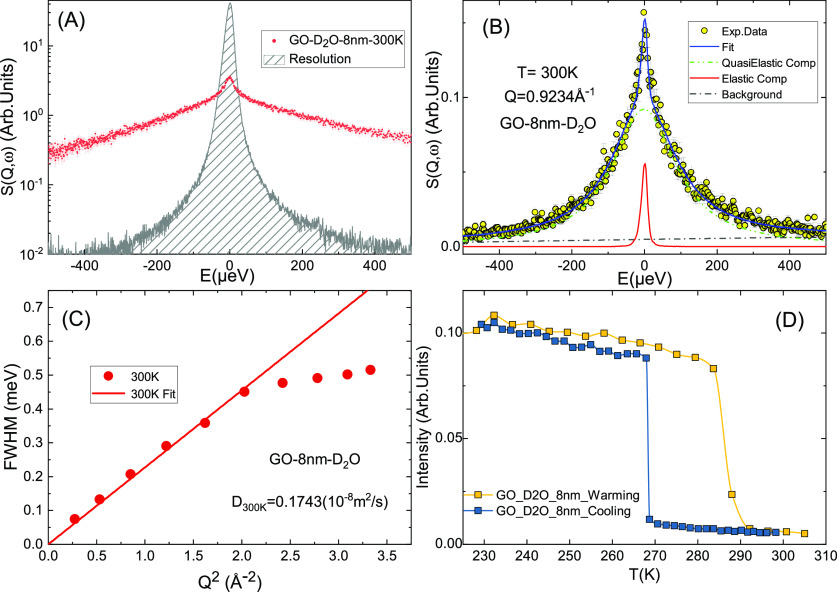
QENS analysis of 8 nm GOs in D_2_O dispersions. (a) Normalized QENS spectra for 8 nm GOs in D_2_O at 300 K, covering the full Q range; (b) an example of model-independent analysis of QENS data at Q = 0.9234 Å^−1^; (c) full width at half maximum (FWHM) vs Q^2^, fitted linearly for smaller Qs to acquire the diffusion coefficient at 300 K; and (d) the elastic scattering intensity plotted as functions of temperature for warming and cooling process of the sample.

To further investigate the dynamical mechanisms behind ice nucleation in GO aqueous dispersions, we performed QENS experiments on GOs in H_2_O and D_2_O, as well as pure water, above and approaching the ice formation temperature. QENS has proved to be a prevailing tool to study the dynamics of water molecules on the timescale of pico- to nano-seconds,^27^ since the hydrogen atoms have much larger incoherent scattering cross section compared to other atoms present in the GO aqueous dispersions. The normalized QENS spectra from IRIS are shown in Figs. 2(a) and 3(a) for GOs in H_2_O and D_2_O, respectively. At 300 K, the central peaks for both samples display obvious broadenings from the resolution functions, indicating large motions of H atoms in the liquid state samples. At 260 and 265 K, the central peaks are very close to the resolution function, indicating ice formation in the samples. Note that the central peak at 265 K is slightly broader than that of 260 K, implying it is the crystallization temperature, which can be clearly observed from the elastic scattering intensity measured on IRIS in Fig. 2(d).

Figure 2(b) demonstrates the QENS analysis of the 8 nm GOs in H_2_O dispersion at 300 K. The measured dynamic structure factor $S_{m}\left(Q,\omega \right)$ can be expressed as $S_{m}\left(Q,\omega \right)=S_{H}\left(Q,\omega \right)⊗R\left(Q,\omega \right),$^28^ where $R\left(Q,\omega \right)$ denotes the instrumental resolution. $S_{H}\left(Q,\omega \right)\;\mathrm{is}$ the self-dynamic structure factor that can be written as a model-independent expression, consisting of elastic and quasi-elastic scattering components, which can be represented by a delta function and a Lorentzian function, respectively, $S_{H}\left(Q,\omega \right)=A_{0}\left(Q\right)\delta \left(\omega \right)+\;(1-A_{0}\left(Q\right))L\left(Q,\omega \right)+B\left(Q,\omega \right),$ where $A_{0}\left(Q\right)$ represents the ratio of elastic scattering, also known as the elastic incoherent structure factor (EISF), $B\left(Q,\omega \right)$ represents the instrumental background, and $L\left(Q,\omega \right)=\frac{1}{\pi }\frac{\Gamma (Q)}{\Gamma^{2}\left(Q\right)+\omega^{2}}$ is a Lorentzian function, also called Cauchy or Breit-Wigner distribution. Here, Γ(Q) denotes the half width at half maximum (HWHM) of the Lorentzian function.

In Fig. 2(c), the fitting parameter full width at half maximum (FWHM), 2Γ(Q), at two different temperatures 300 and 265 K was plotted as functions of Q^2^. At lower Q values, the FWHMs increase linearly with Q^2^, following the DQ^2^ law^29^ derived from the well-known Fick's law and under the condition of Brownian motion,^30^ $S_{\mathrm{inc}}\left(Q,\omega \right)=\frac{1}{\pi }\frac{DQ^{2}}{\omega^{2}+{\left(DQ^{2}\right)}^{2}}$. Therefore, one can directly calculate the diffusion coefficient D from the slope of the linear fitting 2ℏD in the Q range of 0.52–1.42 Å^−1^, which is 0.1086 and 0.1904 (10^−8^ m^2^/s) for 265 and 300 K, respectively. The D value obtained at 265 K is much lower than that at 300 K, suggesting a slower diffusive process at low temperatures and the motion of H-atoms within water molecules has been suppressed when approaching the ice formation temperature [from Fig. 2(d), 265 K is right above the critical temperature for ice formation in the sample]. The diffusion coefficient at 300 K is much smaller than that of bulk water, 0.2411 (10^−8^ m^2^/s) (calculated from an online tool, https://dtrx.de/od/diff/), implying that the dynamics of water was altered by the addition of GOs. At higher Qs, the FWHM values deviate from the DQ^2^ law, implying that the diffusive motions of hydrogen atoms within the samples give smaller contribution to the dynamics at smaller length scale.

Figure 2(d) shows the elastic scan results of GOs with two different sizes in H_2_O and compares the results to pure water, giving the change of elastic scattering intensity with temperature. All samples show thermal hysteresis (TH); that is, the melting temperature in the warming process is ∼25 K higher than the crystallization temperature in the cooling process. The crystallization temperature of 8 and 11 nm GOs in H_2_O is about 260 K, slightly lower than that of pure water (∼265 K). The same plot for the elastic scan results from Emu is shown in Fig. S2 in Ref. 34. A similar crystallization temperature and TH can be found for the GO/H_2_O samples with different concentration. One may note that the crystallization temperatures in QENS experiments are higher than previously reported values^12^ and our TR-SAXS results due to different experimental setups. Specifically, different sample cells were used in the TR-SAXS and QENS experiments. In the SAXS experiment, we used a silica capillary; while in the QENS experiment , we used aluminum cylindrical cans with annular 0.25-mm gap. Different containers provide different interfacial environments for hydrogen bonding network in water, which will result in different crystallization temperatures.

To investigate whether there are differences in the effect of GOs on the dynamics of H_2_O and D_2_O, similar QENS experiments were performed on the sample of 8 nm GO in D_2_O dispersion, with the same mass fraction as the GO/H_2_O sample. From the calculated proportion of scattering cross sections of GOs in these two samples, respectively (see Ref. 34), it is safe to consider the QENS signals just represent the scattering from the water molecules in the samples, and neglect the contribution from GOs. Figure 3 shows the QENS measurement and analysis on the sample of 8 nm GO in D_2_O dispersion. Similar to the GO/H_2_O samples shown in Fig. 2, a model-independent fitting agrees with the measured QENS data of 8 nm GO with D_2_O satisfactorily. In Fig. 3(c), the diffusion coefficient of the D_2_O regulated by GOs is calculated as 0.1743 (10^−8^ m^2^/s), fitted in the same Q range of 0.52–1.42 Å^−1^, slightly lower than that of the GO/H_2_O sample. In Fig. 3(d), the elastic scattering intensity demonstrates a similar thermal hysteresis (TH) effect. The crystallization temperature of the GO/D_2_O sample (∼268 K) is slightly higher than that of the GO/H_2_O sample (∼260 K). The melting temperature is also higher than that of the GO/H_2_O sample (∼288 K vs ∼280 K). The fitting parameter A_0_(Q), known as the EISF, of both GO/H_2_O and GO/D_2_O samples is shown in Fig. S1 in Ref. 34. The EISFs for both samples at 300 K are very small, close to 0, indicating that in the range of the spectrometer, most of the fraction of atoms is mobile. Comparing to the EISF of the GO/H_2_O sample, that of the GO/D_2_O sample has a slightly larger value. Combining with the fact that the FWHM values of the GO/D_2_O sample are smaller than the GO/H_2_O sample, the motions of water molecules in the GO/D_2_O sample are determined to be slower than that in the GO/H_2_O sample. This result is also consistent with the diffusion coefficient values shown in Figs. 2 and 3 that the D value of the GO/D_2_O sample is smaller compared to the GO/H_2_O sample.

**FIG. 4.**
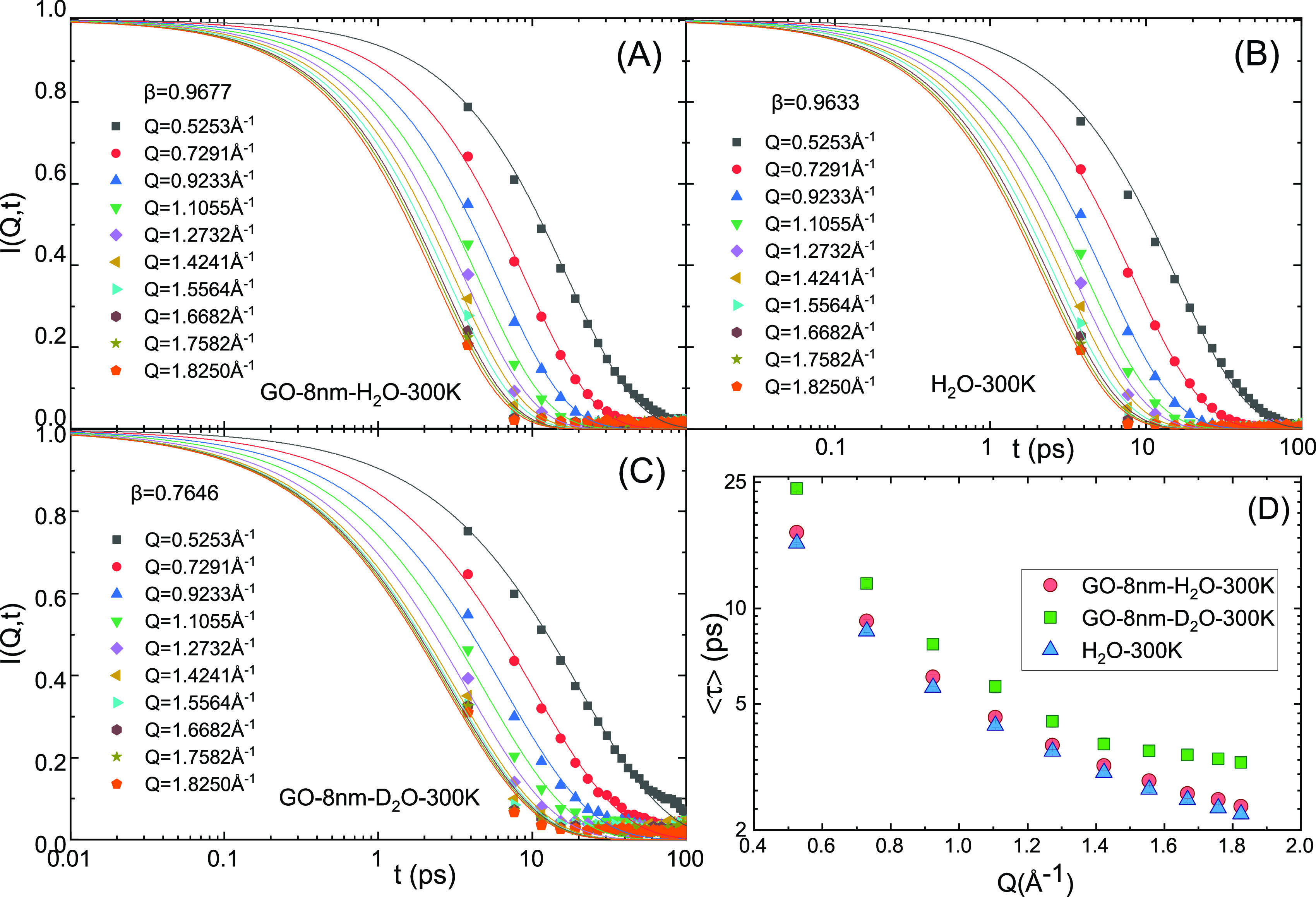
[(a)–(c)] The ISFs of 8 nm GOs in H_2_O, D_2_O, and pure H_2_O fitted with KWW equation (solid lines) at 300 K, respectively, and (d) comparison of the average translational relaxation time ⟨τ⟩ vs Q plots of pure water, GOs in H_2_O, and D_2_O dispersions at 300 K.

The intermediate scattering function (ISF) I(Q,t), referred to as the density–density correlation function, is a primary tool to unravel the relaxational dynamics of water molecules within the samples. The ISF can be calculated by dividing the inverse Fourier transform of the measured QENS spectra $S_{m}(Q,\omega )$ with the inverse Fourier transform of the resolution function R(Q,w). In this study, the ISFs of 8 nm GOs with H_2_O and D_2_O and pure water (H_2_O) were analyzed using a Kohlrausch–Williams–Watts (KWW) law, which has been widely used to describe various dynamical behaviors, such as relaxation, viscosity, friction, gas dynamics, and heat transfer processes,^31^

$$I(Q,t)=f(Q)\;\text{exp}\;[-{\left(\frac{t}{\tau (Q)}\right)}^{\beta }],$$ (1)

where f(Q) is a Q-dependent amplitude, β is the stretch exponent (0 < β < 1), and τ(Q) represents the relaxation time. A Q-independent average relaxation time ⟨τ⟩ is defined as

$$\left\langle \tau \rangle =\frac{\tau }{\beta }\Gamma (\frac{1}{\beta }\right),$$ (2)

where Γ is the Gamma function. In Figs. 4(a)–4(c), the ISFs of the above three samples were fitted according to Eq. (1) at 10 Q values at 300 K, respectively. The stretch exponent β can be considered as Q-independent in the Q range of measurements and can be obtained by fitting curves at all 10 Q values together.^27^

Figure 4(d) shows the average relaxation time ⟨τ⟩ calculated according to Eq. (2) of the above three samples at 300 K plotted as functions of Q. Interestingly, the fact that ⟨τ⟩ of the GO/D_2_O sample is much larger than that in the H_2_O samples at all 10 Q values is consistent with the analysis in the energy domain, which shows that the diffusion coefficient D is smaller for GOs in D_2_O than for GOs in H_2_O. This result indicates that the water molecules in GO/D_2_O sample have slower relaxational dynamics than that in GO/H_2_O sample even at room temperature. However, our results from the elastic scattering [Figs. 2(d) and 3(d)] indicate that the GO/D_2_O sample crystallizes at a higher temperature than the GO/H_2_O sample due to its lower free-energy barrier for ice nucleation. Considering possible hydrogen bonds between the ice nucleus and hydroxy groups on the basal plane of GOs,^32^ our results imply that the effect of GOs in D_2_O leads to lowering of the free-energy barrier for water molecules to form ice nucleation more than that of GOs in H_2_O.

## CONCLUSION

In summary, we experimentally observe a transient intermediate state in ice nucleation through time-resolved SAXS, which only appears in the 11 nm GO aqueous dispersion sample. QENS enables us to study the dynamics of water molecules in GO aqueous dispersions and we compared them with that of pure water. Through elastic scattering intensity, we observe thermal hysteresis (TH) in all samples, which is a characteristic feature shared by all AFPs^16^ and GO aqueous dispersions.^32^ From the analysis of the QENS data in the energy domain, the diffusive process of water molecules is shown to be slower at lower temperatures in GO aqueous dispersions, and therefore, the motion of H-atoms within water molecules has been suppressed during the ice formation, which is consistent with the elastic scattering results. Furthermore, the diffusion coefficient D is smaller for water molecules in GO/D_2_O dispersion than that of water in GO/H_2_O dispersion, implying that the dynamics of water molecules in the D_2_O sample is slower. Meanwhile, the elastic scattering results show that the GO/D_2_O dispersion crystallizes at a higher temperature, indicating that its free-energy barrier is reduced by GO nanosheets more than that of GO/H_2_O dispersion. In time domain analysis, it is observed that the addition of GOs in water results in slightly slower relaxational dynamics, and D_2_O with GOs has much slower dynamics than H_2_O with GOs, which is consistent with the energy domain analysis results. The mechanism of GOs regulating ice nucleation can be understood from the aspect of dynamics: the dynamics of water molecules in GO aqueous dispersion can be suppressed by GO nanosheets, while the addition of GOs above a critical size (11 nm) reduces the interfacial free-energy barrier between water and ice. The result of two competing effects upon addition of GOs leads to ice formation at a higher temperature when the size of GO is larger than a critical size. Since GO nanosheets can mimic the AFPs in controlling ice formation,^16^ the results from this study also serve as a preliminary model for the mechanisms of AFPs in controlling ice formation, from the dynamics point of view.

## AUTHORS' CONTRIBUTIONS

S.Z., J.H., X.L., and Z.W. contributed equally to this work.

## Data Availability

The data that support the findings of this study are available from the corresponding author upon reasonable request. The data that support the findings of this study are openly available in ISIS at DOI: 10.5286/ISIS.E.RB1920374, Ref. 33.
